# Dorsal Metacarpal Artery Flap: An Underrated Workhorse Flap for Reconstruction of Dorsal Finger Defects

**DOI:** 10.7759/cureus.11251

**Published:** 2020-10-29

**Authors:** Leon Alexander

**Affiliations:** 1 Plastic and Reconstructive Surgery, Sheikh Khalifa Medical City, Abu Dhabi, ARE

**Keywords:** dorsal finger defects, dorsal metacarpal artery flaps, perforator flap, reverse flow flap, distally based hand flap

## Abstract

The dorsal metacarpal artery (DMCA) flap is a versatile option in the armamentarium of the reconstructive hand surgeon, especially for resurfacing dorsal finger defects where the flap options are limited. The flap fulfills most of the reconstructive principles such as “to replace like with like” and is simple, reliable, and easy to harvest with minimal donor site morbidity. In this report, we discuss the case of a 37-year-old male patient who presented with a partially healed wound over the right middle finger. Several variations of the DMCA flap have been described in the literature, and these are briefly described here along with their applications and vascular basis.

## Introduction

There are not many options for resurfacing dorsal finger defects when compared to the myriad options available for volar finger defects. The existing options for dorsal finger defects are either unreliable as they are close to the zone of injury (dorsal transposition or rotation flaps, distally based turnover flaps) or entail a two-stage procedure (reverse cross-finger flap) or are complex and involve the transfer of mismatched palmar glabrous skin (neurovascular island flaps) [1]. However, with the recent advances in microsurgery, free flap transfer offers an attractive but complex solution. The literature is replete with reports of the dorsum of the hand being a recipient area for flaps rather than being used as a donor site. To overcome the paucity of reconstructive options for dorsal finger defects, the dorsal metacarpal artery (DMCA) flaps offer a viable and attractive option.

Quaba and Davison first described the DMCA perforator (DMCAP) flap in 1990 as a distally based perforator flap [2]. The Maruyama flap (1990) is a variation of the DMCA flap wherein it is raised as a reverse flow or distally based DMCA flap (reverse dorsal metacarpal artery flap or RDMA flap); here the DMCA is ligated proximally and is included in the flap along with its two venae comitantes [3]. The Quaba flap is based on the perforator present at the level of the neck of the metacarpal about 0.5-1 cm proximal to the metacarpophalangeal (MCP) joint and just distal to the juncturae tendinum. Another variation described in the literature includes the extended RDMA flap based on the terminal connection between the dorsal metacarpal system and dorsal branches of the digital artery at the mid-proximal phalanx level [1-3].

In this report, we present the case of a 37-year-old male patient and discuss all variations of DMCA flaps described in the literature and their applications for the reconstruction of dorsal finger defects.

## Case presentation

A 37-year-old male, a mechanic by profession and right-hand dominant, presented with a partially healed wound over the right middle finger. The wound had been traumatic at the onset; it had been sustained accidentally more than a month ago, and the patient had consulted several doctors who told him that the wound would heal spontaneously by secondary intention. However, he had concurrent superficial wounds over the right index finger and thumb, which had healed by secondary intention. He was a smoker but had no comorbidities and no prior history of drug allergies or surgeries. The probable cause of this partially healed wound was that the initial wound sustained was too big to allow for normal healing, and it was further complicated by exposed extensor tendon, phalangeal bone, and his smoker status.

**Figure 1:**
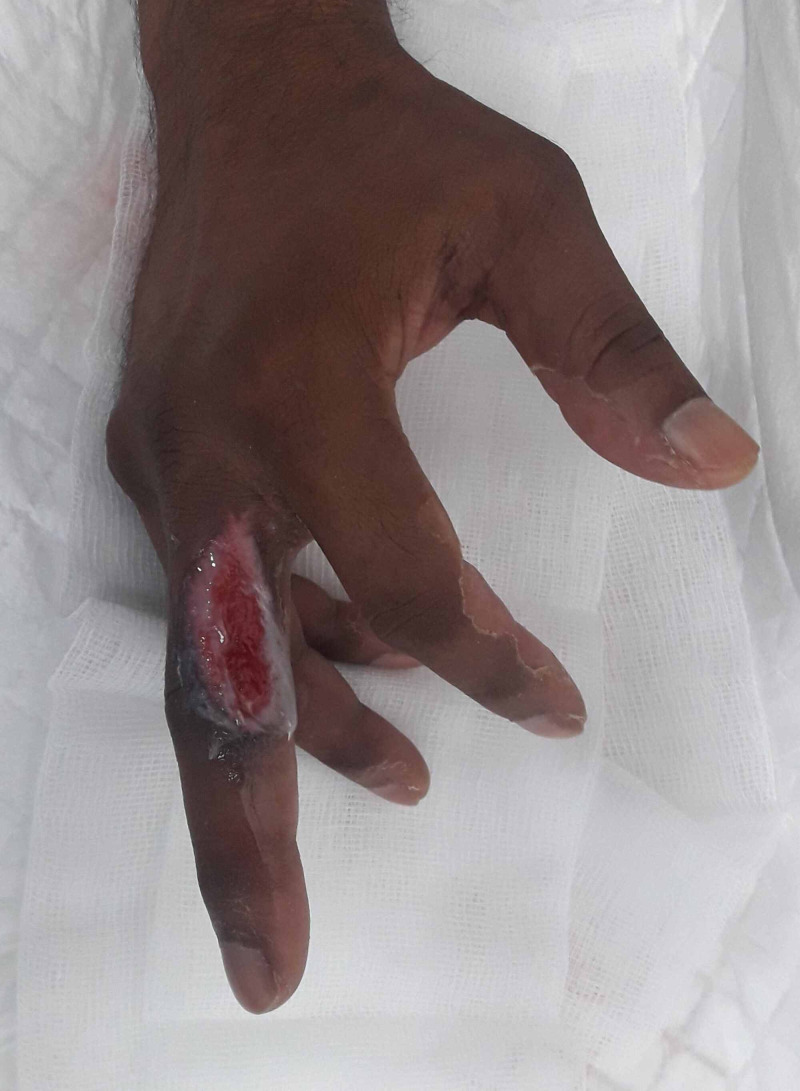
Preoperative view of the defect over the right middle finger Defect over the dorso-radial aspect of the right middle finger at the level of the proximal phalanx and proximal interphalangeal joint

On examination, there was a 4.5 x 1.5-cm wound over the dorso-radial aspect of the right middle finger with exposed extensor tendon and proximal interphalangeal joint (PIPJ) on the superior/dorsal aspect of the wound (Figure 1).

There was no neurovascular deficit distally; his range of motion (ROM) was restricted, with flexion of the PIPJ only possible up to 50 degrees (both active and passive ROM). Vascular examination of the affected hand revealed normal skin color, turgor, warmth, normal distal pulses, normal capillary refill, and the distal sensation was normal when compared with the contralateral hand.

**Figure 2:**
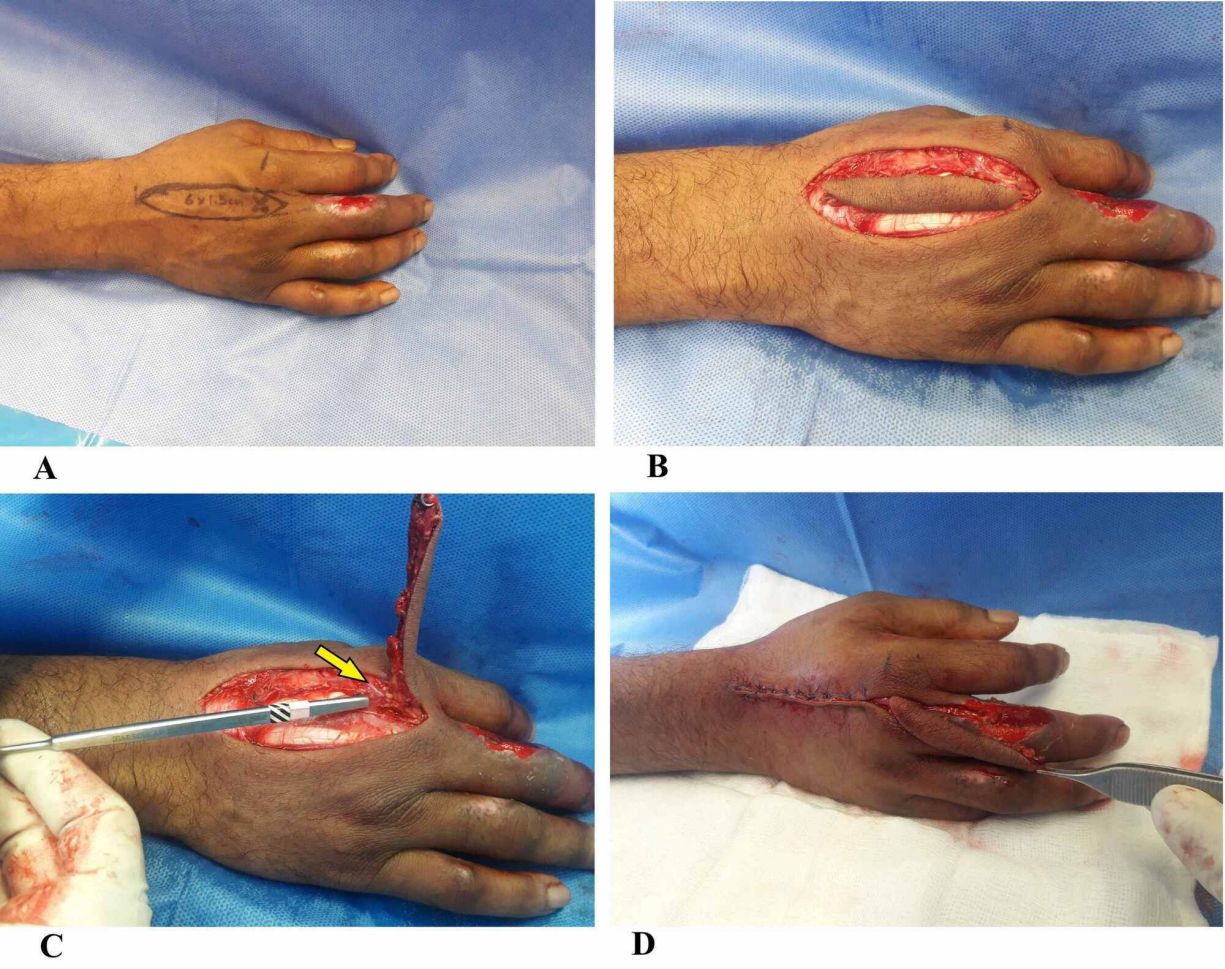
Intra-operative images (A) Intra-operative image of DMCAP flap marking with Doppler perforator. (B) DMCAP flap is islanded. (C) DMCAP cutaneous perforator at the level proximal to second metacarpal head (yellow arrow). (4) The flap is islanded fully and rotated by 90 degrees to reach the defect DMCAP: dorsal metacarpal artery perforator

The flap was raised under tourniquet without limb exsanguination to aid in perforator visualization and dissection. Before prepping, the skin perforator was marked on the second intermetacarpal space using a handheld Doppler probe (Figure 2A), and planning in reverse was done to give an approximate idea of flap design and dimensions, which was then marked. The DMCAP flap was then elevated in the standard fashion as described by Quaba et al.; flap dissection was started proximally. During the flap elevation, superficial veins running along the axis of the flap can be included after ligating proximally, and care must be taken to preserve the paratenon over the extensor tendon. Distally, as the perforator is approached just beyond the juncturae tendinum (Figure 2C), it is important not to skeletonize the perforator so as to preserve the tiny veins that run in the fat surrounding the pedicle and to avoid postoperative venous congestion. Once the flap had been fully islanded based on the metacarpal perforator, the tourniquet was released, hemostasis was obtained, and the flap was allowed to perfuse for about 20 minutes. After this, it was then rotated by 180 degrees; the donor site was closed primarily and the flap was inset (Figure 2D). It is essential that the flap is inset without tension and the donor site closure is also not tight in order to avoid vascular compromise. Dressings were applied, and a volar plaster of Paris slab was used to maintain the wrist and MCP joints in slight extension.

**Figure 3:**
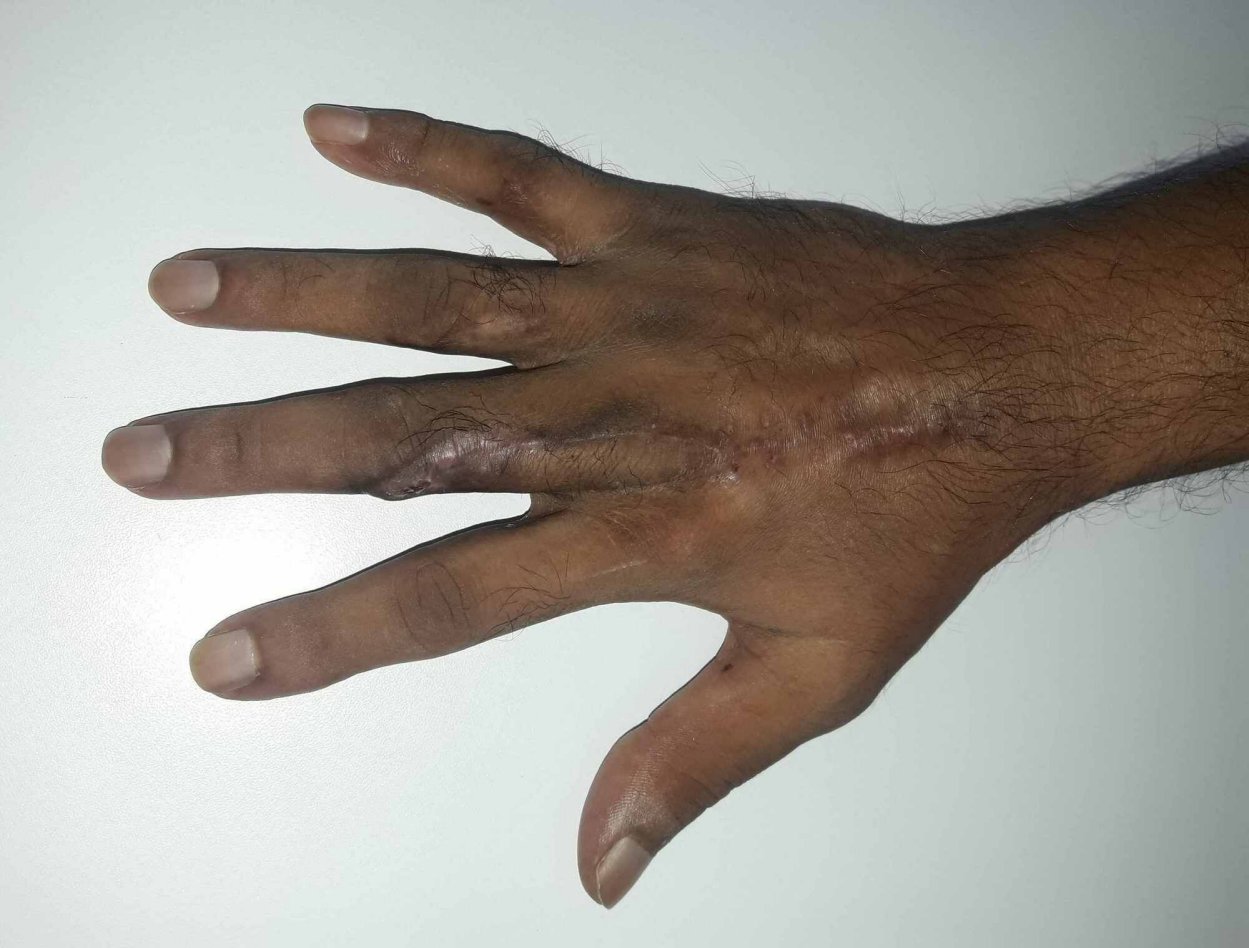
Final outcome The outcome of the affected finger at the six-month follow-up

The postoperative period was uneventful; the patient was discharged the following day with a soft bulky dressing. He attended regular follow-ups and the outcome at six months is shown in Figure 3. The ROM (active) for flexion of PIPJ of the affected finger increased to 100 degrees, and pinch-grip strength was 11.7 (preoperative: 6.7). Quick Disabilities of the Arm, Shoulder, and Hand (QuickDASH) score was 2.3, and the treatment satisfaction visual analog scale (VAS) score was 2/10.

## Discussion

The reconstructive options for dorsal finger defects are limited, and some of the tried and tested options include rotation, advancement, transposition flaps, and hatchet flaps for small defects; for moderate-sized defects, reverse cross finger flaps and adipofascial turnover flaps are an option [4-8]. When large defects are encountered, the use of pedicled abdomen, as well as groin flaps and free flaps have been described [9,10]. The DMCA flaps offer another dimension to the spectrum of flap coverage choices for dorsal finger defects.

**Figure 4:**
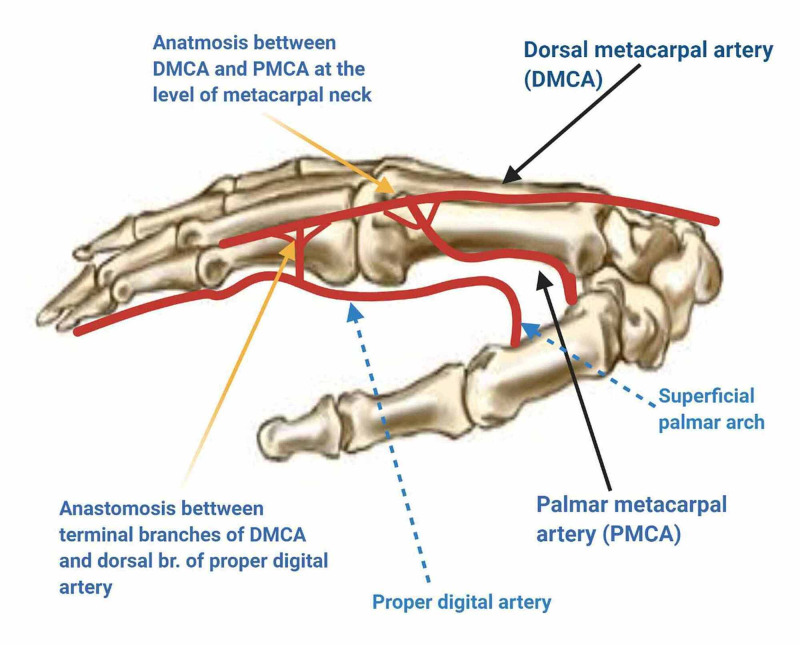
Vascular basis of DMCA flaps

The vascular basis of the DMCA flaps has now been well established and is attributed to the supply by the DMC arterial system or the palmar arterial system through the crucial dorsopalmar anastomosis. This dorsopalmar anastomosis, which is the lifeline of the DMCA flaps, exists at two levels. The proximal connection occurs at the level of the metacarpal neck just distal to the juncturae tendinum between the DMCA and the dorsal perforating branch of the palmar metacarpal artery. The distal connection occurs at the level of mid-proximal phalanx between the terminal branches of the DMCA and the dorsal branches of the proper digital artery (Figure 4).

Studies have also shown that as one proceeds to the ulnar intermetacarpal spaces (third and fourth spaces), the DMCAs may be absent in 17-30% of cases; hence, it is mandatory to use Doppler to locate the presence of DMCA perforator system preoperatively [1,11].

Several variants of the DMCA flaps have been described in the literature. These include the DMCAP flap, extended DMCAP flap, RDMA flap or distally based DMCA flap, extended RDMA (ERDMA) flap, and proximally-based DMCA flap [1,2,12].

**Figure 5:**
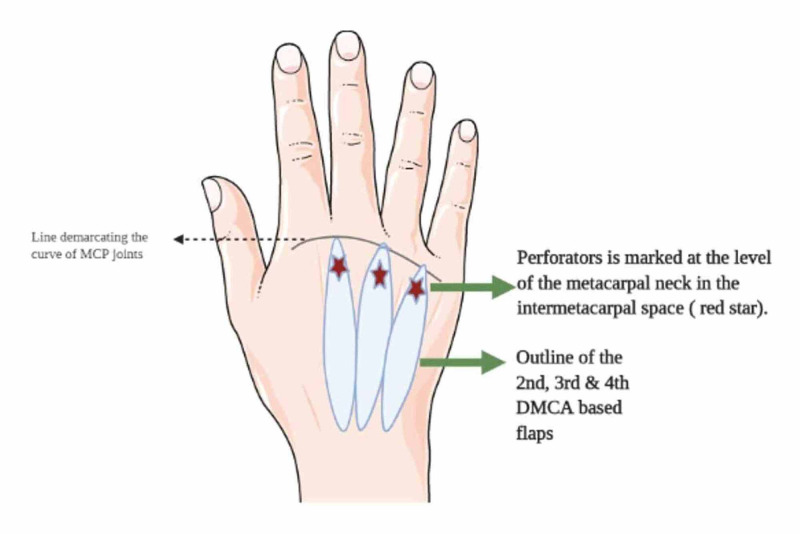
Flap design of DMCAP flap DMCAP: dorsal metacarpal artery perforator

The classic DMCAP flap or the Quaba flap is the most commonly used variant, and it is a perforator flap based on the dominant communicating perforator between the DMCA and palmar arterial system at the level of the metacarpal neck (Figure 5).

It is a perforator flap based on antegrade flow through the perforator and, even though the superficial veins are ligated, flow through the deep veins are undisturbed. Hence it is crucial to avoid skeletonizing the pedicle during flap dissection and to preserve a cuff of subcutaneous tissue around it to preserve venous outflow. When the flap is based on the distal perforator present at the webspace level or mid-proximal phalanx level, it then becomes an extended DMCAP flap with a slightly broader reach and can even reach defects distal to the distal interphalangeal joint (DIPJ) [1-2].

The RDMA flap, also known as the Maruyama flap, is based on the DMCA and is a distally based flap. The flap is centered on the intermetacarpal space, and the proximal limit of the flap is the wrist crease or the extensor retinaculum, and laterally it extends to the outer borders of the adjacent metacarpal bordering the space. It is elevated subfascially over the interossei muscles and after including the DMCA and venae comitantes, which are ligated proximally. The proximal extent of dissection is up to the metacarpal neck where the perforator is visualized and preserved. When the proximal dissection is carried up to the webspace perforator that is preserved, the flap is known as the ERDMA flap [13].

A proximally based DMCA flap has been described for use in finger reconstruction, and it is based on the DMCA proper. It involves harvesting a skin island based on the proximal phalanx of fingers for resurfacing dorsal defects of other fingers or thumb (both volar and dorsal defects). An important point to note when raising this flap is that both the distal and proximal perforators will have to be ligated to ensure optimal mobility of this flap [12].

Indications of the use of DMCA-based flaps and its variants include resurfacing dorsal finger defects up to the DIPJ, finger defects up to the distal phalanx (extended flap variant), and palmar defects proximal to the mid-middle phalanx. Some other atypical uses of this flap include vascularized extensor tendon graft - composite flap to reconstruct missing segments of central slip following post-burn boutonniere deformity [2]. This flap has also been described as a proximally based or distally based vascularized bone flap for reconstruction of carpal bone defects (Kienböck disease) and distal (phalangeal) bony defects, respectively [14,15]. The bilobed second DMCA flap based on the proximal phalanx of the index and middle fingers is a useful and reliable technique for reconstructing composite defects around the MCP joint of the thumb [16]. DMCAP flap has also been used for first webspace reconstruction [17].

When reconstructing defects over the dorsum of the hand and/or fingers, it is essential to bear in mind certain principles. The palmar and dorsal skin are functionally and anatomically different; the dorsal skin is thin, pliable, and mobile to allow for unimpeded and gliding motion of underlying extensor tendons and joints. The aesthetic parameters that must be considered when planning flaps in these areas include the following: color match, texture match, hairiness, final scar location, and development of skin contractures and donor site cosmesis [5].

The DMCA-based flaps fulfill several of the criteria as mentioned above - they have a similar color match, skin thickness, and texture for resurfacing dorsal finger defects. They are reliable and easy to raise, and flaps as large as 27 cm^2^ have been raised. More than one flap can be raised for covering multiple dorsal finger defects. The flap pedicle is arranged predictably, longitudinally along the intermetacarpal spaces with regular anastomosis with palmar metacarpal and proper digital artery in contrast to the palmar circulation where the vessels are many but the arrangement is haphazard, and anastomoses are minimal. The flap can be raised as a vascularized bone or tendon flap to reconstruct bony defects of the phalanges or carpal bones and extensor tendon loss following trauma or burns. The donor site morbidity is acceptable as, in most cases, it can be closed primarily [1,2,5].

These flaps have some drawbacks. Since they are hair-bearing, they are less than ideal for palmar finger defects; they are non-sensate and lead to a conspicuous donor site scar. Venous congestion is another problem with the DMCA flaps due to twisting, kinking, or occlusion of the pedicle, especially the veins. Fortunately, this can be overcome by preserving a generous cuff of subcutaneous tissue around the pedicle during flap harvest, by avoiding tunneling the flap through a skin bridge, and postoperatively removing all constricting sutures, through observation and regular follow-ups [5,18].

## Conclusions

The DMCA flaps and their variants offer another dimension to the reconstructive hand surgeon for resurfacing dorsal finger and hand defects. These flaps are optimal in terms of similar color and texture matches. Moreover, they are reliable and easy to harvest with minimal donor site morbidity.
